# Comparison of cannabinoids in hair with self‐reported cannabis consumption in heavy, light and non‐cannabis users

**DOI:** 10.1111/dar.12412

**Published:** 2016-06-14

**Authors:** Michelle Taylor, Rosie Lees, Graeme Henderson, Anne Lingford‐Hughes, John Macleod, John Sullivan, Matthew Hickman

**Affiliations:** ^1^ MRC Integrative Epidemiology Unit at the University of BristolBristolUK; ^2^ School of Social and Community MedicineUniversity of BristolBristolUK; ^3^ School of PhysiologyPharmacology and NeuroscienceUniversity of BristolBristolUK; ^4^ Centre for NeuropsychopharmacologyDivision of Brain SciencesDepartment of MedicineImperial College LondonLondonUK; ^5^ Alere Toxicology PLc, OxfordUK

**Keywords:** hair testing, cannabinoid, cannabis, sensitivity, specificity

## Abstract

**Introduction:**

*Biological tests of drug use can be used to inform clinical and legal decisions and hold potential to provide evidence for epidemiological studies where self‐reported behaviour may be unavailable or unreliable. We test whether hair can be considered as a reliable marker of cannabis exposure.*

**Methods:**

*Hair samples were collected from 136 subjects who were self‐reported heavy, light or non‐users of cannabis and tested using GC‐MS/MS. Sensitivity, specificity, positive predictive value and negative predictive value were calculated for five cannabinoids (tetrahydrocannabinol [THC], THC‐OH, THC‐COOH, cannabinol and cannabidiol). Samples also were segmented in 1 cm sections representing 1 month exposure and the correlation between amount of cannabinoid detected and self‐reported cannabis consumption tested.*

**Results:**

*All five cannabinoids were detected. Seventy‐seven percent of heavy users, 39% of light users and 0% of non‐users tested positive for THC. The sensitivity of detection of THC was 0.77 (0.56–0.91) comparing heavy cannabis smokers with light and non‐users, whereas the sensitivity of other cannabinoids generally was considerably lower. The positive and negative predictive value of detection of THC were 0.57 (0.39–0.74) and 0.91 (0.82–0.97), respectively. A correlation of 0.52 (P < 0.001) was observed between self‐reported monthly cannabis use and THC.*

**Discussion:**

*Hair analysis can be used as a qualitative indicator of heavy (daily or near daily) cannabis consumption within the past 3 months. However, this approach is unable to reliably detect light cannabis consumption or determine the quantity of cannabis used by the individual.* [Taylor M, Lees R, Henderson G, Lingford‐Hughes A, Macleod J, Sullivan J, Hickman M. Comparison of cannabinoids in hair with self‐reported cannabis consumption in heavy, light and non‐cannabis users. *Drug Alcohol Rev* 2017;36:220‐226]

## Introduction

Self‐reports of cannabis use may be biassed which may lead to problems interpreting evidence on the causes and consequences of drug use 1, 2. Urine tests provide a qualitative measure of recent cannabis use, and are able to detect moderate and chronic heavy use for up to 4 and 24 days later, respectively 3, 4, 5. In contrast the detection window for low level or infrequent cannabis use in urine is comparatively short 6. Cannabinoids are incorporated into the hair shaft from the surrounding blood capillaries, as well as from sebum and sweat that surround the hair shaft 7, 8. Therefore, tests based on hair samples may provide a longer detection window of up to several months and may also measure quantity of cannabis use. As hair grows at a rate of approximately 1 cm per month 9, there is potential for the segmentation of hair samples to provide a measure of when drug use occurred. Finally, hair analysis is quick, sample collection is simple, and it is difficult for the sample to be substituted or forged.

Positive detection of cannabinoids in hair has been documented in several studies 10, 11, 12, 13. Skopp *et al*. 14 provide the only study in which recorded dose is correlated with biomarker. In their study, the amounts of the cannabinoids tetrahydrocannabinol (THC), cannabidiol (CBD) and cannabinol (CBN) were observed to increase in hair in line with increasing cannabis consumption. However, strong correlation between cannabinoids in hair and self‐reported use was not observed, and the study sample (*n* = 12) was small. Stronger evidence, therefore, is required on the sensitivity and specificity of hair analyses for measuring different intensities of cannabis use and how hair analysis could be used in epidemiological and clinical studies.

In this study we examine these issues by comparing sensitivity and specificity for five cannabis metabolites against self‐reported consumption. We also test whether hair could provide a quantitative assessment of the amount of cannabis consumed and if segmenting hair into 1 cm sections (representative of approximately one month of growth) can reliably estimate monthly use.

## Methods

This study was reviewed and approved by South West (Frenchay) Research Ethics Committee (07/H0107/69). A total of 136 participants were recruited from three sources: the University of Bristol, the Bristol Drugs Project and the National Health Service Bristol‐based specialist drug and alcohol service. Heavy cannabis users were recruited from the Bristol Drugs Project 15, a project aimed at providing support and advice to individuals with drug problems. Participants with a range of cannabis use (none to medium) were recruited from the University of Bristol and National Health Service Bristol drug and alcohol service (these participants attended this service for treatment/counselling relating to a drug other than cannabis). Further information on recruitment methods is provided elsewhere 16. Written and informed consent was obtained from all participants. All results were anonymised, and neither participants nor referring services were informed of the results. Hair samples were collected from the posterior vortex of the head, as close as possible to the scalp using scissors. Only the proximal 3 cm hair segment was used, sectioned into either three 1 cm segments or one 3 cm segment, corresponding to approximately the last 3 months of growth.

Samples below 10 mg were subsequently excluded from analysis (as recommended by Alere Toxicology) as below this weight the sensitivity of the test may be compromised.

### Self‐reported cannabis consumption

Participants were asked to report their frequency of cannabis use. Participants who had never tried cannabis or last used cannabis more than 3 months ago were classed as non‐users (i.e. never users/tried in the past but do not use anymore). Based on frequency of cannabis consumption in the last 3 months remaining participants were categorised as light cannabis users (i.e. weekly user or less) or heavy cannabis users (i.e. daily or near daily user).

Additional questions on number of joints/spliffs, pipes or bongs consumed on a typical day were used to generate a continuous measure of monthly cannabis consumption.

### Instrumentation and analysis

Analysis was performed using CTC CombiPal injector attached to a Varian 1200 GC‐MS/MS instrument. The column used was a BPX5 SGE (15 cm × 0.250 × 0.25 µm, SGE Analytical Sciences). Initial column temperature was set at 150 °C for 1 min, and increased to 320 °C. Electron impact mode was used for quantitative analysis. One microlitre elution solvent was injected onto the GC‐MS/MS instrument monitoring four transitions:

1. 374.2 > 308.2THC‐D3, THC‐OH‐D3 and THC‐COOH‐D3
2. 371.2 > 305.2THC, 11‐hydroxy‐Δ9‐THC (THC‐OH) and 11‐*nor*‐9‐Carboxy‐THC (THC‐COOH)
3. 390.2 > 301.2CBD
4. 367.2 > 310.2CBN

### Cannabinoids in hair: sample preparation

Samples were analysed at Alere Toxicology, Cardiff. Two 1 mL methanol washes were performed on the hair with each wash being removed to waste. An additional aliquot of methanol was added and the tubes placed in an unheated ultrasonic bath overnight. Following ultrasonication, the hair sample extract was then placed in a clean test tube. Sodium hydroxide (1 M) was added to the residual hair sample. The sample was placed in a water bath at 80 °C for 30 min. Following cooling the drugs were extracted with chloroform/isopropanol (9:1 v/v) with solid ammonium sulphate (approximately 1 g) added to each sample. After centrifugation, the aqueous layer was aspirated to waste and the solvent layer decanted into the tube containing the original methanol extract. Following addition of 100 μL 0.2 M hydrochloric acid in methanol, the extract was dried under vacuum. The dried extract was reconstituted in ELISA buffer and submitted to solid phase extraction prior to instrumental analysis.

### Solid phase extraction

Solid phase extraction (Waters Oasis MCX μElution plates 30 µm) was used to prepare samples for analysis. Using a vacuum or positive pressure extraction box, the appropriate quantity of solid phase extraction tubes were loaded and conditioned with methanol followed by 0.1 M acetic acid. Each column was loaded with: 0.1 M acetic acid, sample extract is ELISA buffer (0.1 M phosphate buffer pH 7.2), internal standard working solution and the remainder of the column with 0.1 acetic acid; 0.5 mL of sample (hair extract, calibration of quality control sample) was added to each solid phase extraction tube and eluted at a flow not exceeding 1 mL/min. The loaded extract was cleaned using 200 μL 0.1 M acetic acid followed by 200 μL 0.1 M acetic acid:methanol (50:50). The column was dried under vacuum or nitrogen flow for 10 min. Cannabinoids were eluted with hexane and ethyl acetate (80:20 v/v) under gravity and collected into autosampler vials.

### Quantification and validation

The limit of quantification was the lowest calibration standard 0.05 ng/mL, CBD, CBN and THC and 0.004 ng/mL THC‐OH and THC‐COOH. Based on a hair weight of 10 mg, cut offs for samples were applied and were 0.05 ng/mg for CBD, CBN and THC, 0.4 pg/mg for THC‐OH and THC‐COOH. The laboratory was blinded to the self‐reported results. This method has been validated according to the United Kingdom Accreditation Service guidelines for ISO 17025 meeting precision, linearity, quality control, selectivity and specificity criteria. Extraction efficiency for THC has been estimated from external schemes as 36% (where 100% would correspond to complete extraction of drug from the hair), the extraction efficiency of THC COOH has been estimated at approximately 89%.

### Statistics

We undertook two analyses in order to compare and estimate sensitivity and specificity for five cannabinoids (THC, THC‐OH, THC‐COOH, CBD and CBN) and to test whether segmenting hair into 1 cm sections gave reliable results.

First, the sensitivity, specificity, positive predictive value (PPV) and negative predictive value (NPV) were calculated comparing a binary measure of cannabinoid in hair (positive/negative) with four self‐report comparisons: Non‐users compared with light and heavy smokers; non‐users and light smokers compared with heavy smokers; non‐users compared with light smokers and non‐users compared with heavy smokers. Correlation between the above self‐reported measures and a binary measure of THC in hair was also examined.

Second, hair samples that had been cut into 1 cm segments (representing approximately 1 month's growth) were analysed by calculating the correlation coefficient, *r*, between the concentration of cannabinoids in the hair and the continuous measure of self‐reported monthly cannabis consumption. In all above analyses and throughout this report, self‐reported cannabis use was the comparator to the hair analysis.

## Results

Of the 136 participants originally recruited, 105 had hair samples above 10 mg and were included in the analysis. There was evidence for a difference in detection of THC in samples above and below 10 mg. In samples of self‐reported heavy cannabis users, THC was detected in 20 (77%) individuals who provided a hair sample above 10 mg. THC was detected in 6 (32%) self‐reported heavy cannabis users who provided a hair sample below 10 mg (χ^2^ = 8.19, d.f. = 1, *P* = 0.004); thus, low weight samples were dropped from all subsequent analyses.

**Figure 1:**
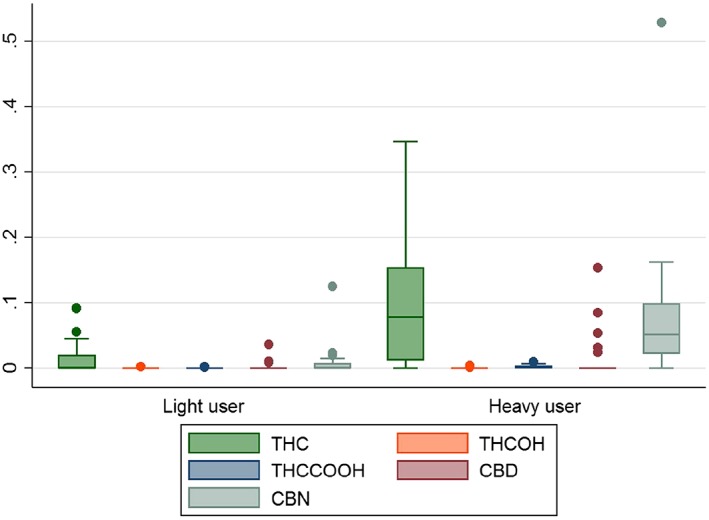
Box and Whisker plot showing amount of cannabinoids detected in the hair of non‐users, light cannabis users and heavy cannabis users, where each cannabinoid is measured in ng/mg. Non‐users were not included in this plot as none of these individuals had any of the cannabinoids detected in their hair.

Over one third of the sample (39%, *n* = 41) were non‐users of cannabis, 36% (*n* = 38) were light users and 25% (*n* = 26) were heavy users. Of those who reported heavy cannabis use, 77% (*n* = 20/26) tested positive for THC. In contrast only 15 (39%) of 38 light users tested positive for THC and none of 41 non‐users tested positive (Table 1). In the sample as a whole, participants smoked an average of 32.3 spliffs/joints per month (s.d. 57.44) (heavy smokers $\bar{x}$ = 116, s.d. = 64.10; light smokers $\bar{x}$ = 23.68, s.d. = 20.29). Measured concentrations of cannabinoids in hair for light and heavy cannabis users are shown in Figure 1.

**Table 1:** Descriptive data of cannabinoids detected using hair testing by self‐reported cannabis smoking status

		Self‐report				
Cannabinoid a		Non‐user b	Light user c	Heavy user d	χ^2^	*P* value
**THC**	**Positive**	0 (0%)	15 (39%)	20 (77%)	43.38	<0.001
	**Negative**	41 (100%)	23 (61%)	6 (23%)		
	**Total**	41	38	26		
**THC‐OH**	**Positive**	0 (0%)	1 (3%)	5 (19%)	11.97	0.003
	**Negative**	41 (100%)	37 (97%)	21 (81%)		
	**Total**	41	38	26		
**THC‐COOH**	**Positive**	0 (0%)	4 (11%)	14 (54%)	34.31	<0.001
	**Negative**	41 (100%)	34 (89%)	12 (46%)		
	**Total**	41	38	26		
**Cannabidiol**	**Positive**	0 (0%)	(11%)	5 (19%)	7.80	0.020
	**Negative**	41 (100%)	34 (89%)	21 (81%)		
	**Total**	41	38	26		
**Cannabinol**	**Positive**	0 (0%)	11 (29%)	19 (73%)	41.64	<0.001
	**Negative**	41 (100%)	27 (71%)	7 (26.9%)		
	**Total**	41	38	26		

a Cut off values to determine positive hair test result: THC, CBD and CBN = 0.05 ng/mg; THC‐OH and THC‐COOH = 0.4 pg/mg.

b Non‐users are those who have never used cannabis or have not used cannabis in the last 3 months.

c Light smokers are those who use cannabis 6 or less times per week.

d Heavy smokers are those who use cannabis more than 6 times a week.

THC, tetrahydrocannabinol.

### Cannabinoid detection in hair

The sensitivity and specificity were calculated for each of the five cannabinoids. When comparing heavy cannabis smokers with light smokers and non‐users, the sensitivity of detection of THC and CBN in hair was 0.77 (0.56–0.91) and 0.73 (0.52–0.88), respectively, whereas the sensitivity of other cannabinoids alone was considerably lower: THC‐OH = 0.19 (0.07–0.39), THC‐COOH = 0.54 (0.33–0.73) and CBD = 0.19 (0.07–0.39) (Table 2).

**Table 2:** Sensitivity, specificity, PPV and NPV for five cannabinoids detected in hair analysis comparing a range of self‐report measures

Cannabinoid	Frequency of cannabis use a, b, c	Sample size	Sensitivity (95% CI)	Specificity (95% CI)	PPV (95% CI)	NPV (95% CI)	Correlation coefficient (r)	*P* value
**THC**	Heavy and light versus non‐users	105	0.55 (0.42–0.67)	1.00 (0.91–1.00)	1.00 (0.90–1.00)	0.59 (0.46–0.70)	0.57	<0.001
	Heavy versus light and non‐users	105	0.77 (0.56–0.91)	0.81 (0.71–0.89)	0.57 (0.39–0.74)	0.91 (0.82–0.97)	0.53	<0.001
	Heavy versus non‐users	81	0.77 (0.56–0.91)	1.00 (0.91–1.00)	1.00 (0.83–1.00)	0.87 (0.74–0.95)	0.82	<0.001
	Light versus non‐users	79	0.40 (0.24–0.57)	1.00 (0.91–1.00)	1.00 (0.78–1.00)	0.64 (0.51–0.76)	0.50	<0.001
**THC‐OH**	Heavy and light versus non‐users	105	0.09 (0.04–0.19)	1.00 (0.91–1.00)	1.00 (0.54–1.00)	0.41 (0.32–0.52)	0.20	0.040
	Heavy versus light and non‐users	105	0.19 (0.07–0.39)	0.99 (0.93–1.00)	0.83 (0.36–0.99)	0.79 (0.69–0.86)	0.33	<0.001
	Heavy versus non‐users	81	0.19 (0.07–0.39)	1.00 (0.91–1.00)	1.00 (0.48–1.00)	0.66 (0.53–0.78)	0.36	0.003
	Light versus non‐users	79	0.03 (0.01–0.14)	1.00 (0.91–1.00)	1.00 (0.03–1.00)	0.53 (0.41–0.64)	0.12	0.302
**THC‐COOH**	Heavy and light versus non‐users	105	0.28 (0.18–0.41)	1.00 (0.91–1.00)	1.00 (0.82–1.00)	0.47 (0.36–0.58)	0.36	<0.001
	Heavy versus light and non‐users	105	0.54 (0.33–0.73)	0.95 (0.88–0.99)	0.78 (0.52–0.94)	0.86 (0.77–0.93)	0.56	<0.001
	Heavy versus non‐users	81	0.54 (0.33–0.73)	1.00 (0.91–1.00)	1.00 (0.77–1.00)	0.77 (0.64–0.88)	0.65	<0.001
	Light versus non‐users	79	0.11 (0.03–0.25)	1.00 (0.91–1.00)	1.00 (0.40–1.00)	0.58 (0.43–0.66)	0.24	0.033
**Cannabidiol**	Heavy and light versus non‐users	105	0.14 (0.07–0.25)	1.00 (0.91–1.00)	1.00 (0.66–1.00)	0.43 (0.33–0.53)	0.25	0.012
	Heavy versus light and non‐users	105	0.19 (0.07–0.39)	0.95 (0.88–0.99)	0.56 (0.21–0.86)	0.78 (0.69–0.86)	0.22	0.025
	Heavy versus non‐users	81	0.19 (0.07–0.39)	1.00 (0.91–1.00)	1.00 (0.48–1.00)	0.66 (0.53–0.78)	0.36	0.003
	Light versus non‐users	79	0.11 (0.03–0.25)	1.00 (0.91–1.00)	1.00 (0.40–1.00)	0.56 (0.43–0.66)	0.24	0.033
**Cannabinol**	Heavy and light versus non‐users	105	0.47 (0.34–0.40)	1.00 (0.91–1.00)	1.00 (0.88–1.00)	0.58 (0.43–0.66)	0.51	<0.001
	Heavy versus light and non‐users	105	0.73 (0.52–0.88)	0.86 (0.77–0.93)	0.63 (0.44–0.80)	0.91 (0.82–0.96)	0.57	<0.001
	Heavy versus non‐users	81	0.73 (0.52–0.88)	1.00 (0.91–1.00)	1.00 (0.82–1.00)	0.85 (0.72–0.94)	0.79	<0.001
	Light versus non‐users	79	0.29 (0.15–0.46)	1.00 (0.91–1.00)	1.00 (0.72–1.00)	0.60 (0.48–0.72)	0.42	<0.001

a Non‐users are those who have never used cannabis or have not used cannabis in the last 3 months.

b Light smokers are those who use cannabis 6 or less times per week.

c Heavy smokers are those who use cannabis more than 6 times a week.

CI, confidence interval; NPV, negative predictive value; PPV, positive predictive value; THC, tetrahydrocannabinol.

The PPV and NPV were also calculated for each of the five cannabinoids. When comparing heavy cannabis smokers with light smokers and non‐users, testing for THC in this sample produces a PPV and NPV of 0.57 (0.39–0.74) and 0.91 (0.82–0.97) respectively. Conversely, when comparing heavy and light cannabis smokers with non‐users, the PPV and NPV were 1.00 (0.90–1.00) and 0.59 (0.46–0.70) respectively (Table 2).

### Hair segment analysis for cannabinoids

Sixty‐nine of the samples were sectioned into three 1 cm segments. One individual had insufficient hair length for testing in segments relating to month 2 and 3, resulting in 68 remaining individuals. 12% (*n* = 8) of individuals had THC detected in all three sections, with 1% (*n* = 1) having THC‐OH, 3% (*n* = 2) having THC‐COOH, 6% (*n* = 3) having CBD and 10% (*n* = 7) having CBN in all three sections of hair.

For the majority of cannabinoids, the 3 cm section (corresponding to exposure 2–3 months ago) gave the highest percentage of cannabinoid detections. The only exception to this was CBD, where both the 1 cm and 3 cm sections had the highest percentage of cannabinoid detected. The highest correlation of 0.52 (*P* < 0.001) was observed between self‐reported heavy cannabis use and the amount of THC in hair, with all segments showing strong or moderate correlation with the self‐reported information (Table 3).

**Table 3:** Correlation between self‐reported monthly cannabis use and amount of cannabinoid in hair in monthly segments

Cannabinoid^a^	Month^b^	Correlation coefficient (*r)*	*P* value	Range (ng/mg)^c^	Positive (*N*)^c^	Negative (*N*)^c^	Positive (%)	Total sample (*N*)
**THC**	1	0.52	<0.001	0.185	8	61	12	69
	2	0.48	<0.001	0.170	11	57	16	68
	3	0.49	<0.001	0.347	18	50	26	68
**THC‐OH**	1	0.38	<0.001	0.001	1	68	1	69
	2	0.38	0.001	0.002	3	65	4	68
	3	0.42	<0.001	0.004	4	64	6	68
**THC‐COOH**	1	0.38	<0.001	0.004	2	67	3	69
	2	0.36	0.001	0.009	4	64	6	68
	3	0.39	<0.001	0.006	6	62	9	68
**Cannabidiol**	1	0.40	<0.001	0.050	4	65	6	69
	2	0.36	0.001	0.084	3	65	4	68
	3	0.36	0.001	0.068	4	64	6	68
**Cannabinol**	1	0.45	<0.001	0.086	7	62	10	69
	2	0.51	<0.001	0.162	10	58	15	68
	3	0.51	<0.001	0.154	16	52	24	68

a Cannabinoid measured as a continuous measure of ng/mg in hair.

b Self‐reported monthly consumption calculated from participants answer to frequency of cannabis use and number of joints/spliffs smoked in a typical session (mean 32.30, s.d. 57.44).

c Positive and negative numbers calculated by applying cut‐off values reported in methods and Table 1, where range is range of cannabinoids in those who tested positive.

THC, tetrahydrocannabinol.

## Discussion

The sensitivity of THC detection in hair was 77% in heavy cannabis smokers compared to light and non‐cannabis users, but fell to 55% in any cannabis users compared to non‐cannabis users. Other metabolites had lower sensitivity and specificity. The concentration of cannabinoids detected in hair was poorly correlated with reported levels of cannabis consumption. When using THC as a marker to detect cannabis use comparing heavy and light smokers with non‐smokers, the PPV indicates that >90% are true positive values. Conversely, a negative result is more difficult to interpret, with the NPV indicating that <60% with negative results are correctly identified as such.

When segmenting hair into 1 cm sections to assess the detection of cannabinoids in ‘monthly’ segments, the highest percentage of individuals had cannabinoids detected in the sections corresponding to exposure three months previous (with CBD being the only exception). Here, we would expect more uncertainty in relation to low level use, and results suggest that segmenting hair into sections to obtain a measure of use over time is likely to be unreliable.

### Limitations

There are several limitations to this research that need to be considered. First, various types of cannabis are available to users and contain different amounts of cannabinoids. For example, ‘skunk’ cannabis is known to have a THC dried weight content in excess of 20% which is far higher than non‐skunk varieties which are more likely to have a typical THC content between 2% and 8% 17. This makes it challenging to quantify cannabis consumption through hair testing. Furthermore, a cannabis cigarette may contain more or less cannabis, dependent on how the user has rolled and prepared their cigarette. The relative ratios of tobacco compounds used may also significantly alter the incorporation of cannabinoids into hair. Second, not only does THC and its main metabolite, THC‐COOH, have a very low incorporation rate in comparison to other drugs, but their neutral and lipophilic nature mean that they do not easily bind to melanin 18. Third, the lack of an additional biomarker of cannabis use (such as urine testing) makes the interpretation of a specific result difficult for scenarios in which the critical determinant is absolute abstinence from cannabis. If a hair sample tests negative for cannabinoids, it is likely that the subject is not a heavy cannabis user, but it cannot be determined accurately whether the individual has been truly abstinent from cannabis. The availability of data from urine tests would provide a replication and comparison of results, therefore enabling us to determine whether the testing of hair is sensitive enough to detect light but not heavy use.

### Comparison with other literature and implications

This study extends the previously published literature in several ways. First, it demonstrates the use of cannabis hair markers in a larger study than previously reported. Additionally, it covers a range of cannabis consumption, across the categories of no, moderate and heavy cannabis use. Skopp and colleagues 14 reported that combining results of cannabinoids provided better correlation with self‐reported consumption data than the majority of individual compounds. However, because of the testing procedure here (whereby only hair screened for cannabis was sent for confirmation of THC‐OH, THC‐COOH, CBN and CBD), any amalgamation of the other cannabinoid results would result in the same sensitivity and specificity of the THC metabolite being observed. In comparison with previous research, the sensitivity and specificity of the cannabis hair test are better than the hair test for alcohol 16.

It has previously been reported that THC can be present in the hair of non‐users. THC‐COOH has been proposed as a better indicator of personal use as this metabolite is only formed after cannabis consumption meaning that a positive hair test cannot be the result of external contamination 19, 20. However, in our sample, we did not detect any THC or THC‐COOH in non‐users and THC gave greater sensitivity and specificity compared to THC‐COOH.

The lower sensitivity for detecting any cannabis use is likely to limit the utility of hair testing in general population samples. For example, if we assume that in a general population sample there are 5% heavy cannabis users and 25% light cannabis users (similar to that observed in general population studies 21), the PPV and NPV of the low prevalence population (comparing non‐users against light and heavy users) would be 0.41 and 0.83 respectively. In this scenario a positive result is more likely to be false positive though a negative result is more likely to be a true negative. Conversely, if the proportion of heavy cannabis smokers in a sample was higher (such as from a clinic or court) then the PPV also would be higher.

Our study has identified hair cannabinoids to be a useful test to detect heavy cannabis use; however, this testing method is unreliable when applied to low to moderate frequency cannabis users. Furthermore, we were unable to use hair testing to determine the quantity of cannabis used by an individual in a specified time frame. As a result, the real life application of hair testing for cannabis use is likely to be limited and might not be applicable to epidemiological surveys of the general population.
